# Alternative Pre-mRNA Splicing in Mammals and Teleost Fish: A Effective Strategy for the Regulation of Immune Responses Against Pathogen Infection

**DOI:** 10.3390/ijms18071530

**Published:** 2017-07-15

**Authors:** Ming Xian Chang, Jie Zhang

**Affiliations:** 1 State Key Laboratory of Freshwater Ecology and Biotechnology, Institute of Hydrobiology, Chinese Academy of Sciences, Wuhan 430072, China; zhangjie@ihb.ac.cn; 2 Key Laboratory of Aquaculture Disease Control, Ministry of Agriculture, Institute of Hydrobiology, Chinese Academy of Sciences, Wuhan 430072, China

**Keywords:** transcriptional regulation, alternative splicing, pattern recognition receptors, signaling molecules, pathogens infection, teleost fish

## Abstract

Pre-mRNA splicing is the process by which introns are removed and the protein coding elements assembled into mature mRNAs. Alternative pre-mRNA splicing provides an important source of transcriptome and proteome complexity through selectively joining different coding elements to form mRNAs, which encode proteins with similar or distinct functions. In mammals, previous studies have shown the role of alternative splicing in regulating the function of the immune system, especially in the regulation of T-cell activation and function. As lower vertebrates, teleost fish mainly rely on a large family of pattern recognition receptors (PRRs) to recognize pathogen-associated molecular patterns (PAMPs) from various invading pathogens. In this review, we summarize recent advances in our understanding of alternative splicing of piscine PRRs including peptidoglycan recognition proteins (PGRPs), nucleotide binding and oligomerization domain (NOD)-like receptors (NLRs), retinoic acid-inducible gene-I (RIG-I)-like receptors (RLRs) and their downstream signaling molecules, compared to splicing in mammals. We also discuss what is known and unknown about the function of splicing isoforms in the innate immune responses against pathogens infection in mammals and teleost fish. Finally, we highlight the consequences of alternative splicing in the innate immune system and give our view of important directions for future studies.

## 1. Introduction

In the initiation of innate immune responses against pathogens, pattern-recognition receptors (PRRs) have an essential role in recognizing the conserved pathogen-associated molecular patterns (PAMPs) and triggering immune responses to eliminate the invading microorganisms. In vertebrate, the most characteristic PRRs include Toll-like receptors (TLRs), peptidoglycan recognition proteins (PGRPs), Nucleotide binding and oligomerization domain (NOD)-like receptors (NLRs) and RIG-I-like receptors (RLRs). The activation of these PRRs could initiate transcriptional and nontranscriptional innate immune responses, which tightly controlled signal transduction pathways and even directed the appropriate adaptive response [1,2,3]. These PRRs-triggered responses are also regulated through themselves and through the involvement of intracellular regulators or amplifiers [4].

Alternative splicing is a versatile regulatory mechanism that allows individual genes to generate more than one mRNA isoform, which in many cases encode functionally distinct proteins [5]. In mammals, more than 90% of human genes undergo alternative splicing [6], and alternative splicing is especially prevalent in the nervous and immune systems [7,8,9]. The importance of alternative splicing is underscored by the fact that misregulated alternative splicing can lead to human disease [10], e.g., the generation of *CD44* splice variants can be linked closely with gastric carcinoma tumorigenesis and differentiation, breast cancer development and progression [11,12,13]. Although numerous immunologically relevant genes, such as pro-inflammatory cytokines and chemokines, have been found to undergo alternative splicing [14,15,16], there has been little effort to develop a coherent picture of how alternative splicing might be used as a general mechanism to regulate the function of PRRs and PRRs-mediated innate immune signaling. In recent years, the alternative splicing and immune function of piscine PRRs and their downstream signaling molecules were investigated in our laboratory. In this review, we summarized what is known and unknown about the alternative splicing and the function of splicing isoforms from PGRPs, NLRs, RLRs and their downstream signaling molecules in response to pathogens infection in mammals and teleost fish.

## 2. Alternative Splicing and Immune Function of Peptidoglycan Recognition Proteins

Peptidoglycan recognition proteins (PGRPs) are evolutionarily conserved pattern recognition receptors from insects to mammals, which recognize bacterial PGN and function in antibacterial innate immunity. Insects *PGRP* genes are classified into short (S) and long (L) transcripts. The short PGRPs include *PGRP-SA*, *SB1*, *SB2*, *SC1A*, *SC1B*, *SC2* and *SD*, with short transcripts and 5′-untranslated regions. The long PGRPs include *PGRP-LA*, *LB*, *LC*, *LD* and *LE*, with long transcripts and 5′-untranslated regions. Most PGRPs have one PGRP domain, which is homologous to bacteriophage and bacterial type 2 amidases [17]. Multiple alternative splicing patterns for the *PGRP-LA*, *LB*, *LC* and *LD* genes have been identified in the fruit fly *Drosophila melanogaster* [18]. The functions of *PGRP-LC* isoforms have been well studied. Alternative splicing of variable extracellular domain-encoding exons generates three membrane-bound receptor isoforms, namely *PGRP-LCa*, *PGRP-LCx* and *PGRP-LCy*. Among them, *PGRP-LCx* isoform is required to mediate signals from gram-positive bacteria and purified bacterial peptidoglycan. *PGRP-LCa* and *LCx* are required for the recognition of gram-negative bacteria and bacterial lipopolysaccharide. *PGRP-LCy* may have a minor role in antagonizing the immune response [19,20].

Mammals have a family of four secreted PGRPs named *PGLYRP-1*, *PGLYRP-2*, *PGLYRP-3* and *PGLYRP-4*, respectively. *PGLYRP-2* is an *N*-acetylmuramoyl-l-alanine amidase that hydrolyzes the lactyl bond between the MurNAc and L-alanine in bacterial peptidoglycan [21]. *PGLYRP-1*, *PGLYRP-3* and *PGLYRP-4* are a new class of bactericidal proteins different from currently known antimicrobial peptides in structure, mechanism of action and expression [22,23,24]. A splicing pattern of *tagL* (*PGRPL*) gene was described in the mouse (*Mus musculus*) [25]. The transcription of *TagL-α′*, *TagL-β′* and *TagL-ε′* splice variants starts from the exon I, *TagL-α*, *TagL-γ* and *TagL-δ* from the exon II. The N-terminal portion of all identified proteins is identical. Among them, TagL-α, TagL-α′ and TagL-β′ contain T phage lysozyme homology domain (also known as PGRP domain) on the C terminus. Frame shift occurring in TagL-γ, TagL-δ and TagL-μ results in the lack of PGRP domain. All these splice variants bound gram-positive, gram-negative bacteria and peptidoglycan, which suggest that the binding does not depend on the presence of PGRP domain.

Three members of the PGRP family were cloned in teleost fish. Unlike human PGRPs, *PGLYRP-2* (or *zfPGRP2*), *PGLYRP-5* (or *zfPGRP-SC*) and *PGLYRP-6* (or *zfPGRP6*) from the zebrafish *Danio rerio* have both amidase and bactericidal activities [26]. *zfPGRP6* and *zfPGRP-SC* also function as pattern recognition receptors to mediate signal transduction [27,28]. RNAi-mediated suppression of *zfPGRP6* significantly down-regulated the expression of those genes involved in a Toll-like receptor signaling pathway [27]. *zfPGRP-SC* could mediate multiple intracellular signaling pathways which may connect with each other to form a complex network to regulate not just immune responses but also other processes such as development and apoptosis [28]. The alternative transcripts also exist in fish *PGRP* homologs. The long PGRPs in teleost fish have multiple alternatively spliced variants [29,30]. In comparison to genomic sequences, the splicing patterns of *tnPGRP-L*, *zfPGRP2* and *gcPGRP6* were determined in the spotted green pufferfish (*Tetraodon nigroviridis*), zebrafish (*D. rerio*) and grass carp (*Ctenopharyngodon idella*) [29,30]. These spliced variants were generated from the deletion of the partial exon 2 (*tnPGRP-L2*, *gcPGRP6a* and *gcPGRP6c*), the whole exon 2 (*gcPGRP6d*), partial exon 3 (*tnPGRP-3* and *zfPGRP-L*), the whole exon 3 (*tnPGRP-L4*), or partial exon 2 and the whole exon 3 (*gcPGRP6b*). The functions of most spliced variants were unclear in teleost fish, except that a report showed that *gcPGRP6* splice variants are able to bind microbial PAMPs and inhibit earlier stage growth of intracellular bacteria [30]. Interestingly, although all gcPGRP6 splice variants have an N-terminal signal peptide, immunofluorescence microscopy and Western blotting showed that the splice variants are intracellular proteins, which are different from the gcPGRP6 normal form [30,31].

## 3. Alternative Splicing and Immune Function of Nucleotide Binding and Oligomerization Domain-Like Receptors

Nucleotide binding and oligomerization domain (NOD)-like receptors (NLRs) were cytosolic sensors of microbial molecules, which have been shown to have many different and important roles in inflammatory responses and host defense against microbial pathogens [32,33,34,35], in maintaining immune homeostasis [36], in the control of autophagy [37] and in regulating early embryogenesis and reproduction [38]. Among four subfamilies that were subdivided according to their amino terminal effector domain [39], NLRA and NLRC subfamilies are conserved in mammals and teleost fish. The other two NLRB and NLRP subfamilies were not identified in teleost fish, and NLRP may represent a mammalian expansion of NLR proteins [40].

### 3.1. NLRA Subfamily

The NLRA subfamily includes only one member, the major histocompatibility complex (MHC-II) transactivator (*CIITA*). *CIITA* functions as a master control factor for *MHC class II* genes expression. CIITA contains an N-terminal acidic domain (AD), followed by a region rich in proline, serine and threonine (P/S/T region), a central GTP-binding domain (GBD) and a C-terminal leucine rich repeat domain [41]. Multiple variants and differential splicing patterns were found in mammalian *CIITA*.

Alternative promoter usage: Four isoforms of *CIITA* (*CIITA type I, II, III* and *IV*) were generated by alternative promoter usage. These *CIITA* isoforms are differed only in their N-terminal ends [42]. Of the four different *CIITA* isoforms, human *CIITA type III* corresponds to the previously described form of *CIITA* cDNA [41]. The *CIITA type II* and *IV* use the same ATG which is located 21 bp downstream of the 5′ end of the common nucleotide sequence, and encode the same protein. *CIITA type I* and *III* use the ATG located upstream of the common nucleotide sequence, and generate CIITA proteins with an additional 101 or 24 N-terminal amino acids respectively. The pattern of *CIITA* promoter usage was analyzed by RNase protection assays on the specific transcripts of the endogenous *CIITA* gene, which revealed a strong bias in the selective use of different *CIITA* promoters in the control of both constitutive and inducible expression of *CIITA* [42].

A variety of insertions and/or deletions were seen in the coding region and additional sequences were found at their 3′ ends: In *MHC class II*-positive B cells, *CIITA* cDNA clones showed alternative RNA splicing [43]. CIITA-8 was considered to produce a wild type (wt) protein. *CIITA-2.11* contained an insertion of 479 bp within the coding sequence beginning at the base pair position 596 in wt *CIITA*, and also contained an additional 30 bp at the 3′ end. *CIITA-1.23* contained the 3′ 248 bp of the inserted DNA found in the coding region of *CIITA-2.11* at base pair position 596. DNA sequence analysis indicated that both *CIITA-2.11* and *CIITA-1.23* contained stop codons in all reading frames. CIITA-10 contained a 1 aa insertion at base pair 473, a 49 aa in-frame deletion between base pairs 596 and 744, and a stop codon resulting in a truncated protein of 884 aa instead of 1130 aa. Among these variants, only *CIITA-8* was able to restore *class II MHC* gene expression.

Alternative splice donor site: Defective *MHC class II* expression in an *MHC class II* deficiency patient is caused by ATU *CIITA*, a novel deletion of a splice donor site in the *CIITA* gene [44]. ATU *CIITA* with the lack of 84 nucleotides failed to transactivate *MHC class II* genes and did not display a dominant negative effect on *CIITA*-mediated transactivation of various *MHC class II* promoters.

Exon skipping: In primary cells, two novel splice variants of human *CIITA* were identified [45]. One variant *CIITAΔE7* is devoid of the entire exon 7, which results in the loss of aa 160–209 in the N-terminal part of P/S/T domain of the CIITA protein. *CIITAΔE7* exhibits altered functions toward those chaperons involved in regulating HLA class II assembly and transport.

Intron retention: In K-562 cells, an alternatively spliced transcript of *CIITA* was identified [46]. This variant contains an insertion of 870 bp genomic sequence, which introduces a stop codon at nt 2796 and results in a truncated protein of 932 amino acids rather than 1130. The alternative transcript was not present in Raji cells. Although the alternative CIITA protein is able to associate with the MHC class II promoter and the RFX complex, the transactivation ability of CIITA variant is abolished, compared with wt CIITA.

Different from mammalian *CIITA*, the study on fish *CIITA* was rather limited. Only two reports showed the phylogeny and expression analysis of *CIITA* in channel catfish (*Ictalurus punctatus*) [47,48]. *CIITA* has been referred to as the "master control factor" for the expression of *MHC class II* genes [49]. Interestingly, the deficiency of zebrafish nucleotide-binding oligomerization domain-containing protein 1 (*NOD1*) significantly attenuated the expression of *MHC-II β* and *mhc2dab* [50], which suggested that *NOD1* functioned as a new regulator to drive the expression of *MHC* genes. Further studies are needed to clarify if piscine *CIITA* plays redundant or exclusive roles with *NOD1* in the regulation of the expression of *MHC* genes.

### 3.2. NLRC Subfamily

The NLRC subfamily consists of five members: *NLRC1* (*NOD1*), *NLRC2* (*NOD2*), *NLRC3*, *NLRC4* and *NLRC5*. These NLRCs can function as either positive or negative regulators of inflammatory signaling cascades. Among these members, the homologues of mammalian *NLRC4* were not identified in teleost fish. Here, the alternative splicing and immune function of other four members except *NLRC4* are summarized in this review.

#### 3.2.1. Nucleotide-Binding Oligomerization Domain-Containing Protein 1 and 2

**Figure 1:**
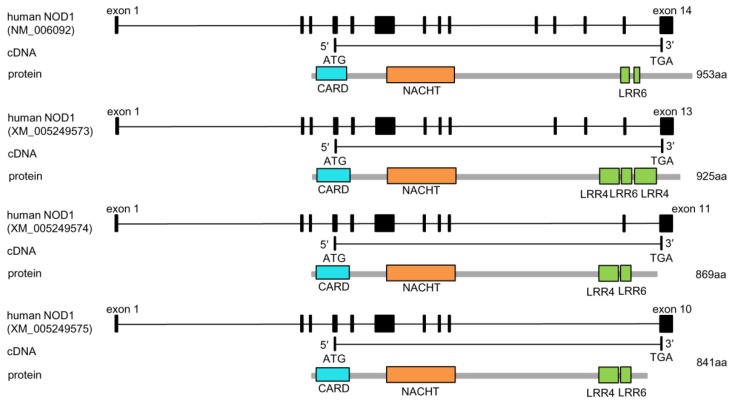
The alternative splicing of human *NOD1*. Exons are indicated as square boxes, and the introns as straight lines. CARD: caspase activation and recruitment domain; NACHT: nucleotide-binding oligomerization domain; LRR: Leucine-rich repeats.

*NOD1* and *NOD2* are the best-characterized members of NLRC subfamily. Significant advances have been achieved regarding the function of *NOD1* and *NOD2* in innate immune responses to bacterial, parasite and viral infections [51,52,53,54]. Although alternative pre-mRNA splicing was only reported for *NOD2* but not *NOD1* in mammals and teleost fish, analysis of human databases revealed the existence of multiple splicing variants of *NOD1* (Figure 1). Different from mammalian NOD1, only one form of NOD1 exists in zebrafish (*D. rerio*) since Western blotting exhibits the single band in the lysates from ZF4 cells and wild type zebrafish using monoclonal anti-NOD1 antibody [50].

The mammalian *NOD2* gene has 12 exons and encodes a protein of 1040 amino acids [55]. At least 8 *NOD2* splicing variants were identified. An abundant NOD2 splice variant lacking exon 3 leads to a predicted 21-kDa short NOD2 protein variant (NOD2-S) with a complete CARD1, a truncated CARD2 domain (54 amino acids) and 10 previously undescribed C-terminal amino acids. Besides NOD2-S, another N-terminally spliced variant NOD2-35 was generated by retention of part of intron 1 and a frameshift. NOD2-35 encoded only the first 25 N-terminal amino acid residues of NOD2, followed by a novel sequence of 10 amino acid residues [56]. A novel alternative promoter and novel first exon of NOD2 are responsible for producing a protein of 1023 amino acids, which is likely to be translated from the first ATG in exon 2 (known as Met28) [57,58]. In addition, five NOD2 variants are generated by alternatively spliced transcripts of the LRR domains [56]. Among these identified variants, NOD2-S interacts with NOD2 and RIP2 and abolishes MDP induced NOD2 self-association, and also functions as a negative regulator of NOD2/RIP2-induced NF-κB activation [59]. Except for *NOD2-S*, the functional significance of most *NOD2* splicing variants is unknown.

Similar to mammalian *NOD2*, the piscine *NOD2* gene undergoes splice variation. In zebrafish (*D. rerio*), a cryptic splice site in exon 1 resulted in a predicted NOD2 molecule with a single CARD but intact NOD and LRR domain [60]. In rainbow trout (*Oncorhynchus mykiss*), two NOD2 transcripts were confirmed by RT-PCR [61]. The shorter transcript of rainbow trout *NOD2* (*trNOD2a*) encodes the normal form. Another transcript named *trNOD2b* had a longer 5′ untranslated region (UTR) and a 65 bp deletion including the normal start codon ATG, resulting in the predicted translation starting from the next downstream ATG. The first CARD domain is incomplete in trNOD2b. The 38 aa deletion in the first CARD domain of trout NOD2 has no significant effect on the induced expression of proinflammatory cytokines including *IL-1β*, tumor necrosis factor-α (*TNF-α*), *IL-6* and *IL-8*, the antibacterial peptide *cathelicidin-2*, a variety of caspases including *caspase-6, -7, -8, -9*, and *type I* and *type II IFN* [61].

#### 3.2.2. NLR Family CARD Domain Containing 3

NLR Family CARD Domain Containing 3 (*NLRC3*) was shown to be a negative regulator, which negatively regulates diverse aspects of host antiviral immunity including *STING*, *type I IFN* and *TLR*-induced NF-κB signaling to attenuate overzealous inflammation following virus infection [62,63]. In addition, *NLRC3* negatively regulates T cell function [64], and also functions as an inhibitor of the mTOR pathways [65,66]. The splicing variants of *NLRC3* were not found either in mammals or fish species. In teleost fish, several studies showed the cloning and expression pattern of *NLRC3* in turbot (*Scophthalmus maximus L.*) [67], rainbow trout (*O. mykiss*) [68], Asian seabass (*Lates calcarifer*) [69], Japanese flounder (*Paralichthys olivaceus*) [70], miiuy croaker (*miichthys miiuy*) [71] and channel catfish (*I. punctatus*) [72,73]. The functions of piscine *NLRC3* were quite unclear at present, although a study showed that zebrafish NLRC3-like, which contains the canonical pyrin (PYD) and NACHT domains but lacks the common LRRs, prevents inappropriate macrophage activation, thereby allowing normal microglia development [74].

#### 3.2.3. NLR Family CARD Domain Containing 5

The role of mammalian *NLRC5* (also known as *NOD27* and *CLR16.1*) in regulating innate and adaptive immune responses has been controversial. The study by Cui et al. showed the negative regulation of *NLRC5* in antiviral signaling and type I *IFN* production [75], but little or no role in regulating *IFN* levels or virus replication from the report of Kumar et al. [76]. The positive regulation of *NLRC5* in *IFN*-dependent or *RIG-I*-mediated antiviral responses was reported in three other groups [77,78,79]. Besides this discrepancy, *NLRC5* has been linked to the *NLRP3* inflamasome and *MHC* class I transactivation [80,81,82,83]. *NLRC5* interacts with *NLRP3* to cooperatively activate the inflammasome [80,81]. *NLRC5* exclusively transactivates *MHC* class I and related genes through a distinctive SXY module [84].

**Figure 2:**
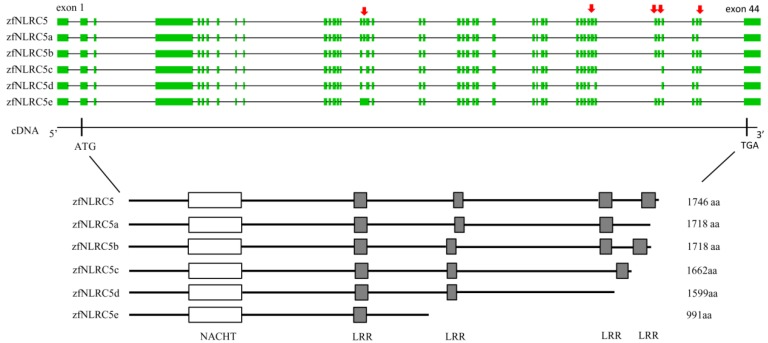
The alternative splicing of zebrafish *NLRC5*. Exons are indicated as square boxes, and the introns as straight lines. GenBank accession numbers for zebrafish *NLRC5* isoforms are: zfNLRC5, AFN73230; zfNLRC5a, AFN73231; zfNLRC5b, AFN73232; zfNLRC5c, AFN73233; zfNLRC5d, AFN73234; zfNLRC5e, AFN73235. NACHT: nucleotide-binding oligomerization domain; LRR: Leucine-rich repeats. The alternatively spliced exons were indicated in the red arrows.

In human *Homo sapiens* [78] and zebrafish *D. rerio* (Figure 2), 5 different splice variants were obtained. All shared a conserved 5′ region but differed in the length of the LRRs. In mammals, the LRR domains of NLR proteins are essential for sensing of their PAMPs and DAMPs. The unusual structure of the NLRC5 LRR domain might thus be indicative for NLRC5 to respond to quite different stimuli than other NLRs [78]. Although the exact biological function of these NLRC5 isoforms has yet to be investigated, our unpublished studies in vivo and in vitro showed the functional difference of zebrafish NLRC5 isoforms in viral infection. Interestingly, our research also showed that zebrafish *NLRC5* normal form is involved in an IFN-independent antiviral response and also functions as a transcriptional regulator of *MHC* class II genes [85], which is different from mammalian *NLRC5*. Further studies are needed to understand the function and the mechanism of *NLRC5* isoforms in response to different pathogens infection.

## 4. Alternative Splicing and Immune Function of Retinoic Acid-Inducible Gene-I-Like Receptors

RLRs are well conserved intracellular PRRs among vertebrates. The RLR family consists of retinoic acid-inducible gene-I (*RIG-I*), melanoma differentiation-associated factor 5 (*MDA5*) and laboratory of genetics and physiology 2 (*LGP2*). RIG-I and MDA5 share similar domain structures, including two N-terminal caspase activation and recruitment domains (CARDs), a distinct DEX/DH box RNA helicase domain and a C-terminal regulatory domain (CTD or RD) [86]. The N-terminal CARD domains facilitate RIG-I and MDA5 interacting with other CARD containing molecules. The central DExD/H-box region with ATP hydrolysis activity is homologous to RNA helicase domain, and involved in dsRNA interactions. The RD domain is crucial for the specific recognition of RNA substrate [86,87]. In mammals, *RIG-I* and *MDA5* function as positive regulators in antiviral innate immunity [88]. The third RLR family member *LGP2*, also known as *Dhx58*, harbors a DExD/H-box helicase domain and a C-terminal RD but lacks any CARDs which functions as a positive [89] or negative regulator [90,91] in *RIG-I*- and *MDA5*-mediated antiviral responses.

**Figure 3:**
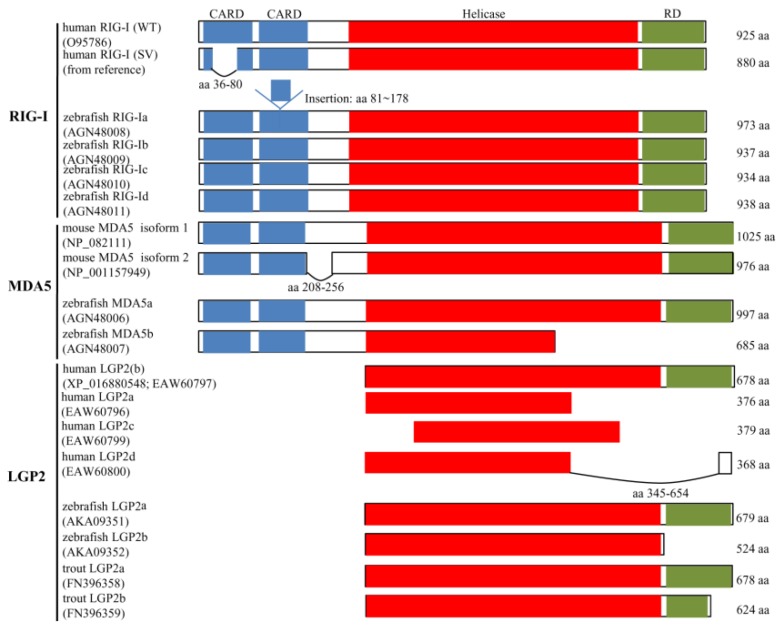
The alternative splicing of mammalian and piscine RLRs. CARD: caspase activation and recruitment domain; Helicase: helicase_insert_domain superfamily; RD: regulatory domain.

In teleost fish, RLRs were first found in 2008 using bioinformatic analysis [92]. *RIG-I*, *MDA5* and *LGP2* genes have been cloned in crucian carp (*Carassius auratus*) [93], common carp (*Cyprinus carpio*) [94], black carp (*Mylopharyngodon piceus*) [95,96], grass carp (*C. idella*) [97,98,99], zebrafish (*D. rerio*) [100,101,102,103], channel catfish (*I. punctatus*) [104], orange spotted grouper (*Epinephelus coioides*) [105,106], Atlantic salmon (*Salmo salar*) [107], rainbow trout (*O. mykiss*) [108], large yellow croaker (*Larimichthys crocea*) [109], green chromide (*Etroplus suratensis*) [110], sea perch (*Lateolabrax japonicas*) [111,112] and Japanese flounder (*P. olivaceus*) [113]. Similar to those orthologs in mammals, piscine RLRs could be spliced at RNA levels, which lead to sequence deletion or insertion in the open reading frame (ORF) (Figure 3).

*RIG-I* gene in zebrafish had four different transcripts. Compared with *RIG-I* from mammalian and other fish species, zebrafish RIG-Ib encodes the normal form. The residues RKPFEIKISFTRVTWPQARRQEVKTEGALQIHRGALDL in RIG-Ia are inserted in the second CARD domain of RIG-I, which shows no sequence homology with any reported RIG-I in other fish species or in mammals [101]. Zebrafish RIG-Ic encodes a protein that lacks the first 189–192 amino acid region just behind the second CARD of RIG-I. Zebrafish RIG-Id encodes a protein that lacks 2 aa just behind the second CARD, however inserts 3 aa within the Helicase domain. Zebrafish *RIG-I* genomic DNA sequence has not yet been completely assembled in the latest version GRCz10 (Genome Reference Consortium Zebrafish Build 10). Different from *RIG-I*, the genomic DNA sequence of *MDA5* is clear in zebrafish. The *MDA5a* gene consists of 16 exons, whereas *MDA5b* lacks partial exon 11, the entire exon 12 and partial exon 13. The C-terminal RD domain is absent for zebrafish MDA5b [100]. Two *LGP2* splicing variants were identified both in rainbow trout (*O. mykiss*) and zebrafish (*D. rerio*) [103,108]. The identified trout *LGP2* cDNA (named *LGP2a*) encodes a protein of 678 aa. Trout LGP2b is 54 aa shorter than LGP2a due to an intron of 1,040 bp retained at the 3′-end region of the ORF, which results in the early termination of translation [108]. The zebrafish LGP2b (DrLGP2b) is a truncated isoform of LGP2a (DrLGP2a). Compared with DrLGP2a, the DrLGP2b lack a regulatory domin (RD) (551–672 aa) at the C-terminal (Figure 3). All sequences of fish RLRs including *RIG-I*, *MDA5* and *LGP2* isoforms refer to transcripts.

In mammals, the function of *RIG-I* splicing variant was reported. The RIG-I SV, lacking a critical part of the first CARD, loses TRIM25 binding, CARD ubiquitination, and downstream signaling ability. Furthermore, RIG-I SV suppresses the RIG-I-mediated IFN-β production through inhibiting the formation of virus-induced RIG-I multimerization and RIG-I-mitochondrial antiviral signaling protein (MAVS) signaling complex [114]. In zebrafish, although the RIG-Ia variant, with 38 amino acids inserted in the second CARD, loses the activity to induce the activation of IFN promoter and protect cells against spring viraemia of carp rhabdovirus (SVCV) infection, RIG-Ia functions as an enhancer in the RIG-Ib/MAVS-mediated signaling pathway [101]. The functions of other two *RIG-I* variants (*RIG-Ic* and *RIG-Id*) are unclear at present. Similar to zebrafish *MDA5a*, the truncated *MDA5b* variant can also induce an antiviral response due to the presence of the intact tandem CARDs [100], which is consistent with the finding in *RIG-I* that the over-expression of the N-terminal CARDs was able to protect cells against virus infection [115]. In addition, *MDA5b* can augment the *IFN* production induced by *MDA5a* and *MAVS* [100]. In teleost fish, most studies showed piscine *LGP2* functions as a positive regulator in antiviral responses [95,96,113,116], except for negative regulation of the antiviral response by *LGP2* from orange-spotted grouper (*E. coioides*) and grass carp (*C. idella*) [105,117]. *LGP2* splicing variants were only identified in zebrafish (*D. rerio*) [103] and rainbow trout (*O. mykiss*) [108]. Trout LGP2a acted as a positive regulator in antiviral responses, whereas LGP2b with a deletion of 54 amino acids at the C terminus RD domain acts as a negative regulator for LGP2a-elicited antiviral signaling by competing for the viral RNA PAMPs [108]. The exact roles of the two zebrafish *LGP2* isoforms involved in viral infection are still unclear [103].

## 5. Alternative Splicing and Immune Function of Downstream Signaling Molecules

### 5.1. Mitochondrial Antiviral Signaling Protein

The mitochondrial antiviral signaling protein (*MAVS*), also known as *CARDIF*, *IPS-1*, *KIAA1271* and *VISA*, is an innate immunity protein that functions downstream of RIG-I-like receptors (*RLRs*) to link RNA virus invasion to the type I interferon (IFN) pathway. Mammalian *MAVS* gene encodes a number of splice variants that have been proposed to negatively regulate *MAVS* signaling. Mammalian *MAVS* gene is encoded by a single gene composed of 6 exons. MAVS 1a (deletion of exon 2), containing a putative CARD domain and a TRAF2-binding motif, interacts with RIP1 and TRAF proteins and functions as an inhibitor against MAVS activation on IFN-β and NF-κB promoters through disrupting RIG-I/MAVS signaling complex formation. MAVS1b (deletion of exon 3) shares the first 97 residues with wt MAVS and 27 aa residues of unknown protein. Different from wt MAVS, which activates both NF-κB and IRF3 pathways, MAVS1b promotes signaling complex formation involving FADD and RIP1 for IFN-β activation. MAVS1c (deletion of exon 6), which encodes 386 aa residues and is a truncated form of MAVS, has no activity on either NF-κB or IRF3 pathway [118]. In addition, translation of mammalian MAVS can also be initiated by two different translation start sites. This alternative internal translation of MAVS results in the production of a shorter variant of 398 amino acids that lacks the CARD domain, and is referred to as miniMAVS which essentially serves as an inhibitor of wt MAVS signaling [119].

Piscine MAVS contains similar protein domains as in mammals, with an N-terminal CARD domain, a central proline-rich region and a C-terminal TM domain [115,120,121,122,123,124]. Except for wt *MAVS*, *MAVS* variant is only cloned in zebrafish (*D. rerio*). This shorter variant named MAVS_tv2, lacking a C-terminal TM domain, is generated from a frame shift due to intron insertion, whose C-terminal 41 aa residues share no sequence similarity to any known proteins in the database [125]. Interestingly, different expression constructs of MAVS_tv2 exhibited the functional differences [125,126]. The EPC cells transfected with ptGFP1-MAVS_tv2 were more resistant to SVCV infection than the control cells transfected with ptGFP1 empty plasmid. In addition, overexpression of MAVS_tv2-FLAG in EPC cells induced the activation of IFN1 and IFN3 promoters. Furthermore, overexpression of MAVS_tv2-FLAG in zebrafish embryos can significantly increase the expression of many antiviral genes such as *IFN1*, *IFN2*, *IFN3*, *mxc* and *rsad2* [125]. All these data suggested the positive regulation of *MAVS_tv2* in the antiviral response. Different from ptGFP1-MAVS_tv2 and MAVS_tv2-FLAG, overexpression of pcDNA-MAVS_tv2 could not affect the *IFN1* activity. On the other hand, overexpression of pcDNA-MAVS_tv2 decreased the activation of *IFN1* promoter and the transcriptional levels of several IFN-stimulated genes induced by *IRF7*, which suggested that *MAVS_tv2* is a negative regulator of *IFN1* by targeting *IRF7* [126]. More studies are needed to make sure the exact function and mechanisms of *MAVS_tv2* targeting in the different signaling molecule of RLRs signaling pathway in response to viral infection.

### 5.2. Stimulator of Interferon Genes

Stimulator of interferon genes (*STING*) (also known as *MITA* or *ERIS*) has been found to be another adaptor protein that links upstream pathogen sensing to downstream *IFN* induction [127,128]. MITA, comprising 5 putative transmembrane (TM) regions, predominantly resides in the endoplasmic reticulum and is able to activate both NF-κB and IRF3 transcription pathways to induce type I IFN [127]. Intensive studies have established the essential role of *STING* in sensing nucleic acids such as the cytosolic double-stranded DNAs and c-di-GMP or c-di-AMP [129,130,131,132,133,134,135,136]. The MITA/TBK1/IRF3 axis has been found to be important in RLRs-mediated and some DNA sensor-mediated antiviral signaling pathways [129,135,137,138]. *MITA* is also reported to be a target molecule for microbial pathogens such as yellow fever virus, dengue virus and hepatitis C virus to escape the innate immune response [139,140,141].

A splice variant of *MITA* (designated as *MRP*) lacking exon 7 was identified in human (*H. sapiens*). The absence of exon 7 resulted in a frame shift, whose putative protein is identical to aa 1-253 of wt MITA at the N-terminal but possesses a unique 30-aa sequence at the carboxyl terminal [142]. Interestingly, *MRP* plays a role as a negative regulator in *MITA*-induced activation of the *IFN* signaling pathway by sendai virus infection and cyclic diguanylate treatment, but enhanced the herpes simplex virus type 1 (HSV-1) induced *IFN* response [142]. In addition, a recent study showed that *MRP*, despite its inability to trigger *IRF3* activation, could restrict hepatitis B virus (HBV) replication in vitro and in vivo via the activation of NF-κB pathway [143].

In teleost fish, *MITA* was only reported in crucian carp (*C. auratus*) [93], zebrafish (*D. rerio*), fathead minnow (*Pimephales promelas*) [144], orange spotted grouper (*E. coioides*) [145] and grass carp (*C. idella*) [146]. Similar to mammalian *MITA*, piscine *MITA* activates IFN response via MITA-TBK1-IRF3 signaling pathway [93,145], and is also the target of virus to escape the innate immune response [146]. Our unpublished data showed that zebrafish *MITA* variant is generated by Exon skipping. Zebrafish MITA variant is identical to aa 1–244 of wt MITA at the N-terminal but possesses a unique 17-aa sequence at the carboxyl terminal. The function of piscine *MITA* variant is unclear at present, and need to be further investigated.

### 5.3. TRAF Family Member-Associated NF-kappaB Activator (TANK) Binding Kinase 1

TANK binding kinase 1 (*TBK1*) is a serine/threonine-protein kinase, and acts as a critical player in the regulation of the immune response to bacterial and viral challenges, inflammatory responses, the insulin signaling pathway and autophagy [147,148,149,150,151]. As the pivotal role of *TBK1* in various immunobiological and immunopathological events, its activity must be tightly regulated to effectively control pathogen infection and maintain immune homeostasis. *TBK1* activity is regulated in a variety of ways including phosphorylation, ubiquitination, kinase activity modulation and prevention of functional TBK1-containing complexes formation [152]. The splice variant of *TBK1* was only reported in human (*H. sapiens*) and mouse (*M. musculus*), and named as TBK1s. Excision of exons 3–6 of *TBK1s* results in translation from the second ATG and leads to an in-frame deletion of the kinase domain (amino acids 1–234). Different from TBK1, TBK1s can bind to RIG-I through its coiled-coil domain, and negatively regulates virus-triggered IFN-β signaling pathway by disrupting the interaction of RIG-I and MAVS [153].

The function of *TBK1* in regulating IFN-I pathway was studied in teleost fish. The *TBK1* (CiTBK1) from grass carp (*C. idella*) participates in the antibacterial and antiviral immune responses in different manners. After LPS stimulation, *CiTBK1* triggered *IFN-I* activation which was independent of *IRF3*/*IRF7*. Post GCRV challenge, *CiTBK1* mediated *IFN-I* response mainly by *IRF7* not *IRF3*. In addition, *CiTBK1* negatively regulated PGN-induced *IRF3*, *IRF7*, *IFN-I* and *Mx1* immune response [154]. Similar to piscine *MAVS* and *MITA*, piscine *TBK1* is also targeted by viruses as a major negative regulatory target to decrease the IFN response and facilitate viral replication. Spring viremia of carp virus (SVCV) P protein functions as a decoy substrate for cellular TBK1, leading to the reduction of IRF3 phosphorylation and suppression of IFN expression [155]. In zebrafish (*D. rerio*), a TBK1-like transcript (TBK1L), containing an incomplete S_TKc domain and lacking UBL_TBK1_like domain, was cloned. Overexpression of zebrafish TBK1L negatively regulated the production of IFN and IFN-stimulated genes through RLRs-MAVS-TBK1 pathway [156]. In addition, a study showed that the *TBK1* from large yellow croaker (*L. crocea*) can be regulated by *Nrdp1*, an E3 ubiquitin ligase, and was involved in the immune defense against the pathogen infection [157].

### 5.4. Interferon Regulatory Factor 3

The transcription factor IRF3 plays a critical role in the regulation of IFN production following virus infection. The TBK1 and the inhibitor of NF-κB kinase-ε (IKKε) can phosphorylate IRF3. Phosphorylated IRF3 subsequently dissociates from the adaptor protein, and then forms a homo- or heterodimer with other transcriptional factors before translocating into the nucleus to induce transcription of IFNs [158,159]. In mammals, multiple *IRF3* isoforms have been characterized. Different from the normal form of *IRF3*, an additional exon located between exon 2 and 3 was designated 3a, which encoded a distinct 20-amino-acid N terminus of IRF-3 [160]. Due to lack half of the DNA binding domain found in IRF-3 normal form, human IRF-3a spliced isoform failed to bind with ISRE sequences, and negatively regulated the transcriptional activity of IRF3 [161]. The second spliced isoform IRF3-nirs3, which lacked 127 amino acids in the regulatory domain (RD) of IRF3 normal form, was found in human hepatocellular carcinoma (HCC) cells. IRF3-nirs3 overexpression impaired significantly the expression of IFN-β, and was benefiting for viral replication [162]. The third spliced isoform IRF3-CL was ubiquitously expressed in various cell lines including liver and tumor cell lines. Compared with *IRF3* normal form, the additional 16 nucleotides upstream of exon 7 in *IRF3-CL* generated a frame shift, which gave rise to a distinct carboxyl-terminus without the autoinhibition element (AIE) domain. *IRF3-CL* functioned as a competitive inhibitor of *IRF3* [163]. Two novel *IRF3* spliced isoforms, *Int2V1* and *Int2V2*, were cloned from pheochromocytoma tissue. The expression of *Int2V2* was higher than *Int2V1* in a variety of tissues and cell lines examined, except for in HepG2 cell line [164]. The functions of *Int2V1* and *Int2V2* are unclear at present. In addition, five other splicing isoforms of *IRF3*, namely *IRF-3b*, *-3c*, *-3d*, *-3e* and *-3f*, were identified in human cells. These *IRF3* isoforms were generated by exon deletions, and attenuated the transactivation activity of *IRF3* [165].

Although *IRF3* has been reported in multiple fish species such as miiuy croaker (*m. miiuy*) [166], grass carp (*C. idella*) [167], tilapia (*Oreochromis niloticus*) [168], half-smooth tongue sole (*Cynoglossus semilaevis*) [169], European eel (*Anguilla anguilla*) [170], large yellow croaker (*L. crocea*) [171], Japanese flounder (*P. olivaceus*) [172] and crucian carp (*C. auratus*) [93], splicing variants of *IRF3* have still not been well studied. Analysis the zebrafish database and the results from our sequencing of *IRF3*, at least 3 splicing variants are found in zebrafish (Supplement Figure S1). Interestingly, *IRF3c* cloned by us may generate two ORFs, which both encode *IRF3*. The first ORF encodes 125 aa, which are corresponding to 1~125 aa of IRF3a (Supplement Figure S2). The second ORF encodes 337 aa, which is 87.24 and 88.08% identities with IRF3a and IRF3b, respectively (Supplement Figure S3). It is interesting to know the exact function of these piscine *IRF3* variants.

### 5.5. Interferons and Their Receptors

Interferons (IFNs) play a major role in the defense against virus infection in vertebrates. There are three types of IFNs in mammals. Type I IFNs consist of seven classes: *IFN-α*, *IFN-β*, *IFN-ε*, *IFN-κ*, *IFN-ω*, *IFN-δ*, and *IFN-τ*. Type II IFN consists of *IFN-γ* only. The type III IFNs or IFN-λs, which are comprised of three intron-containing members and are known as *interleukin (IL)-29*, *IL-28A* and *IL-28B* [173,174]. Type I IFNs transduce intracellular signals through the common receptor *IFNAR1/2* [175], Type II IFN by recognizing cell surface receptor *IFNGR1/2* [176], type III IFNs by *IL-28R1/IL-10R2* receptor complex [177]. Mammalian *IFNAR* genes encode multiple isoforms that contribute to the complexity of the functional receptor. The major ligand-binding subunit *IFNAR-2* exists in 3 mRNA splice variants including 2 transmembrane isoforms (*IFNAR-2b* and *IFNAR-2c*) and a soluble isoform (*IFNAR-2a*) [178]. Among the 3 *IFNAR-2* isoforms, *IFNAR-2c* is recognized as the functional one, whereas *IFNAR-2b* is unable to perform signal transduction [179] and may act as a dominant negative regulator of IFN responses [180]. Although lacking the transmembrane and intracytoplasmic domain, the third isoform *IFNAR-2a* is still capable of binding IFNs, and functions as an important regulatory factor of type I IFNs activity [181]. Different from *IFNAR-2*, only one *IFNAR-1* isoform with transcriptional capacity was identified in normal cells [182,183].

Among the three IFN families, only type I and II *IFN* genes were identified and well documented in teleost fish [184]. In contrast with the single exon *IFN* genes in reptiles, birds and mammals, fish type I *IFN* genes are consisting of five exons and four introns [185,186]. The alternative splicing of piscine type I IFN genes has been reported in rainbow trout (*O. mykiss*) [187,188]. The splicing isoforms of trout *IFN1* are generated by the absence or retention of the first and/or second introns and the usage of different ATG transcription start site. In rainbow trout, there are three ATG transcription start sites at the 5′ end of the transcript. Secretory *sIFN1* with the retention of the first and second introns uses the second ATG for transcription initiation. Intracellular *iIFN1a* with the retention of the first intron uses the third ATG for transcription initiation. Intracellular *iIFN1b* without the first and second introns uses the first ATG for transcription initiation. Similar to secretory *IFN1*, the intracellular *IFN1* isoforms possess antiviral activity and are able to activate cellular antiviral pathways [188]. Furthermore, membrane bound and intracellular IFN receptors, named as *mIFNAR2* and *iIFNAR2*, are generated by alternative splicing. *mIFNAR2* conserves the first intron in the coding region and uses the first ATG for transcription initiation. *iIFNAR2* without intron retention uses the second ATG for transcription initiation. The intracellular IFNs induce the expression of antiviral genes and STAT phosphorylation through intracellular IFN receptors [188].

In addition to *IFN-γ*, the *IFN-γrel* molecule, which has duplicated from the *IFN-γ* gene, is generally accepted as a second member of the type II IFN family in teleost fish [184]. Two *IFNγ* spliced isoforms are found in catfish (*I. punctatus*) and medaka (*Oryzias latipes*) [189,190]. *IFNγ2a* and *2b* were cloned from the gonadal cDNA of medaka. *IFNγ2a* exhibited ubiquitous expression, while *IFNγ2b* was only expressed predominantly in female germ cells than males. The alternative splicing of *IFNγ* in medaka is steroid-dependent [190]. Two *IFNGR1* isoforms were also cloned in zebrafish (*D. rerio*) and goldfish (*Carassius auratus L.*) [191,192]. In vitro binding studies indicated that goldfish IFNGR1-1 bound to IFNγ1 but not IFNγ2, while the IFNGR1-2 bound to IFNγ2 [191].

## 6. Conclusions and Prospects

Those studies on PRRs and PRRs-mediated innate immune signaling highlight the pivotal role of innate immune in controlling pathogen infection. The importance of alternative splicing in finely regulating the immune homeostasis is now beginning to be appreciated. Indeed, those PRRs (*PGRPs*, *NLRs* and *RLRs*) and their downstream signaling molecules (*MAVS*, *MITA*, *TBK1*, *IRF3*, *IFNs* and their receptors) are alternatively spliced. In addition, those splicing isoforms are cross-modulated for PRRs-triggered responses in either a cooperative or an antagonistic manner. In mammals, plenty of work has been done to identify the sequences, proteins and mechanisms by which splicing are regulated in the PRRs-mediated signaling pathways, or splicing variants regulate the function of PRRs-triggered innate immune responses. Several intriguing and important aspects of the splicing and immune function for those members from NLRC and NLRP subfamilies are still unclear and therefore remain to be done in future research. In teleost fish, the alternative splicing of *IFN* and *IFN receptors* can lead to a functional intracellular IFN system, which acts as a novel defense to combat viral infection. Further studies will be needed to determine whether the intracellular IFN system function as a widespread mechanism in vertebrate evolution.

**Figure 4:**
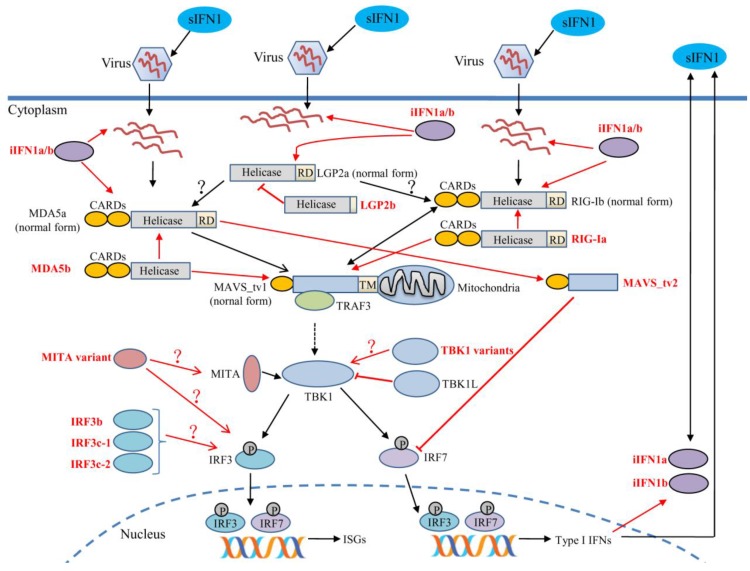
The alternative splicing and immune function of RLRs-mediated signaling pathways in response to viral infection in teleost fish. *RIG-I*, *MDA5*, *LGP2*, *MAVS*, *MITA*, *TBK1*, *IRF3* and *IFN1* undergo alternative splicing. *RIG-Ia* functions as an enhancer in the *RIG-Ib*/*MAVS*-mediated signaling pathway, *MDA5b* as an enhancer in the *MDA5a*/*MAVS*-mediated signaling pathway, *LGP2b* as a negative regulator for *LGP2a*-elicited antiviral signaling. *MAVS_tv1* cooperates with *RIG-Ib* in a positive feedback loop and enhances *RIG-Ib*-mediated antiviral signaling, whereas *MAVS_tv2* synergizes with *MDA5a* and enhances *MAVS_tv2*-mediated antiviral signaling. *MAVS_tv2* may also function as a negative regulator of *IFN1* by targeting *IRF7*. The function of those splicing isoforms of *MITA*, *TBK1* and *IRF3* is still unclear at present, and need to be further investigated, which were indicated by the arrows with “?”. Importantly, fish possess a functional intracellular IFN system. The cross-regulation among excellular and intracellular IFN system function as a positive feedback loop in RLRs-MAVS signaling pathways. In the signaling schematics, the signaling pathways mediated by splicing isoforms are marked with red arrows, black arrows for normal form or wild type of PRRs signaling molecules, bidirectional arrows for the interaction between different PRRs signaling molecules. The broken arrow indicates that the direct interaction need to be confirmed.

Although many splicing isoforms of most PRRs as well as the downstream signaling molecules have currently been identified in zebrafish, the exact mechanisms of splicing isoforms and the specific ligand(s) recognized by different PRR variants remain poorly understood in teleost fish. Our previous studies show clearly that splicing isoforms *RIG-Ia*, *MDA5b*, *MAVS_tv2*, *iIFN1a* and *iIFN1b* function as positive regulators for RLRs-triggered responses, whereas *LGP2b* and *TBK1L* as negative regulators for RLRs-triggered responses in teleost fish (Figure 4). Much work is needed to understand how different variants of NLRs family affect distinct signaling molecules, and also to understand the physiological and pathological significance of alternative splicing. In addition, the cross-regulation among different PRR variants may further endow them with the ability to properly respond to a large variety of invading pathogens. The interplay effect between PRR variants and/or other immune pathways on the host immune defense responses also requires further investigation.

Given the fact that aberrant splicing is known to contribute to defects in immune function and that the expressions of splicing isoforms have been linked to the ability to induce a protective immune response to pathogens or the ability of the pathogen to evade host immune response, there is no doubt that alternative splicing has strong effects on the immune system. The knowledge of alternative splicing of PRRs and PRRs-mediated innate immune signaling will shed new light on the pathogenesis of inflammatory diseases and provide important clues for the control of pathogens infection.

## Supplementary Materials

Supplementary materials can be found at www.mdpi.com/1422-0067/18/7/1530/s1.
