# Supplementary material for: Alternative Pre-mRNA Splicing in Mammals and Teleost Fish: A Effective Strategy for the Regulation of Immune Responses Against Pathogen Infection
Source: Int J Mol Sci. 2017 Jul 15;18(7):1530. doi: 10.3390/ijms18071530 (PMC5536018; doi:10.3390/ijms18071530)
Supplement: Supplementary file 1 [file ijms-18-01530-s001.pdf]

Figure S1: Multiple alignment of the nucleotide sequences of zebrafish IRF3a, IRF3b and IRF3c. Identical residues are identified by stars, whereas similar residues are identified by single dots.

```
IRF3a          ATTGTTCATTGTTATTTTTTAATGTAGCCAATAAACAAATAAATCTTATTTTAAAGTGCT
IRF3b          ------------------------------------------------------------
IRF3c          ------------------------------------------------------------

IRF3a          AAATAAACAACGACAGGCCTGACACACACGAAAACACATTTTAATCGTCTGTTGTTTACG
IRF3b          ------------------------------------------------------------
IRF3c          ------------------------------------------------------------

IRF3a          TTCTTTTTTAATGGTCGTCTCCGCCATCTTGTGGTCATGCAGGGTAAAATAACTTACGGT
IRF3b          ------------------------------------------------------------
IRF3c          ------------------------------------------------------------

IRF3a          GACGTGTGTAATGACGCGAAAAGGGGAAATCGAAACTGATATGGGTTTCCCGAGTAATGT
IRF3b          ------------------------------------------------------------
IRF3c          ------------------------------------------------------------

IRF3a          TTAAAACCTGATCGTAAAGCACAGTCTGCTGAATAAAGCTCAGTATTAAAACAACATTGA
IRF3b          ------------------------------------------------------------
IRF3c          ------------------------------------------------------------

IRF3a          CAAGCAGTTCTGTCTACTTACATCTCTTACATCTGAGGAAGAGCTCGCTGAAAACACACG
IRF3b          ------------------------------------------------------------
IRF3c          ------------------------------------------------------------

IRF3a          AGGCCACAAACAACTGTCGCTTCTGTTTGCAAGGTCCTGTCTATTGATGAATGTTGTGCT
IRF3b          ------------------------------------------------------------
IRF3c          ------------------------------------------------------------

IRF3a          GTATAGTCTGACTGCATCATGACTCAAGCAAAACCGCTGTTCGTGCCCTGGTTGTATGAA
IRF3b          ------------------ATGACTCAAGCAAAACCGCTGTTCGTGCCCTGGTTGTATGAA
IRF3c          ---------------ACCATGACTCAAGCAAAACCGCTGTTCGTGCCCTGGTTGTATGAA
                                 *****************************************

IRF3a          CAAATCCAGAGTGGACGTTATCCTGGAGTATGCTGGAAAAATGAAGACTGTACCCAGTTC
IRF3b          CAAATCCAGAGTGGACGTTATCCTGGAGTATGCTGGAAAAATGAAGACTGTACCCAGTTC
IRF3c          CAAATCCAGAGTGGACGTTATCCTGGAGTATGCTGGAAAAATGAAGACTGTACCCAGTTC
               ************************************************************

IRF3a          AGCATTCCCTGGAAACACGCTTTGAGACAGGACTCCAACAGCGACGATGTGCTCATATTC
IRF3b          AGCATTCCCTGGAAACACGCTTTGAGACAGGACTCCAACAGCGACGATGTGCTCATATTC
IRF3c          AGCATTCCCTGGAAACACGCTTTGAGACAGGACTCCAACAGCGACGATGTGCTCATATTC
               ************************************************************

IRF3a          AAGGCGTGGGCTCAGACTAGCGCTGCTGGTGATGGCAGATTGAATGGAGATCCGTCTGTG
IRF3b          AAGGCGTGGGCTCAGACTAGCGCTGCTGGTGATGGCAGATTGAATGGAGATCCGTCTGTG
IRF3c          AAGGCGTGGGCTCAGACTAGCGCTGCTGGTGATGGCAGATTGAATGGAGATCCGTCTGTG
               ************************************************************

IRF3a          TGGAAGAGAAACTTTCGCAGTGCTCTTCGCGCTAAAGGCTTCAAAATGATATCTGACAAA
IRF3b          TGGAAGAGAAACTTTCGCAGTGCTCTTCGCGCTAAAGGCTTCAAAATGATATCTGACAAA
IRF3c          TGGAAGAGAAACTTTCGCAGTGCTCTTCGCGCTAAAGGCTTCAAAATGATATCTGACAAA
               ************************************************************

IRF3a          AAGAATGATGGCGCCGATCCACATAAAGTCTATCAGTTTCCCTCCGATCCTCATTCTGCA
IRF3b          AAGAATGATGGCGCCGATCCACATAAAGTCTATCAGTTTCCCTCCGATCCTCATTCTGCA
IRF3c          AAGAATGATGGCGCCGATCCACATAAAGTCTATCAGTTTCCCTCCGATCCTCATTCTGCA
               ************************************************************

IRF3a          GCTTCAGGATCTGAGGGCTCTCAAGAAACTGAT---------------------------
IRF3b          ------------------------------------------------------------
IRF3c          GCTTCAGGATCTGAGGGCTCTCAAGAAACTGATTGATACCGGTAAATATCTGGAAAATTT


IRF3a          ------------------------------------------------------------
IRF3b          ------------------------------------------------------------
IRF3c          CCGGAACGACTTTACCGGTATATTAAAAAAGCGCTGTTCTCACAGGCGAGGACATTACGG

IRF3a          ------------------------------------------------------------
IRF3b          ------------------------------------------------------------
IRF3c          AAATTTTCCGGAAAAGAGCATTCACACATCCATTCCAAAATACCGGATTACTTTCGCATG


IRF3a          ------------------------------------------------------------
IRF3b          ------------------------------------------------------------
IRF3c          TCGAGATGTTCATAATATGTGCGTGTGCTGGCGCTCACAGGCTGTTTCAAAGGCACACGC


IRF3a          -----------ATTTCACCACATCTGTATGTGGATAATGTGTTCACAGCATCATTTGATT
IRF3b          ------------------------------------------------------------
IRF3c          AAAGCTTAAAGATTTCACCACATCTGTATGTGGATAATGTGTTCACAGCATCCCTTGATT
```

```
IRF3a        TGGACCAACATTTAACTGGACTACACCTCCAGGACCCATCAGCACGAGTCCATATCATTT
IRF3b        -----------------------------------------------GTCCATATCATTT
IRF3c        TGGACCAACATTTTACTGGACTAAACCTTCAGGACCCATCAGCACACGTCTATAGAATTT
                                                            *** ***  ****

IRF3a        TGAATCCAAATGACTTATATGTGGATCCAGGAACCCAGATATACGATTTTCAAGCAGTGG
IRF3b        TGAATCCAAATGACTTATATGTGGATCCAGGAACCCAGATATACGATTTTCAAGCAGTGG
IRF3c        TGAATCCAGATGACTTATATGTGGATCCAGGAACCCAGATACTCGATTTTCAAGCAGTGG
             ******** ********************************  *****************

IRF3a        TCCCTATTGAAGCCATATCCTCCTCAGATGAAGGTGCTGTCGGTGGTTTGGGACAGATTT
IRF3b        TCCCTATTGAAGCCATATCCTCCTCAGATGAAGGTGCTGTCGGTGGTTTGGGACAGATTT
IRF3c        TCCCTATTAAAGCCATATCCTCCTCAGATGAAGGTGCTGTCGGTGGTTTGGGAAAAATTT
             ******** ******************************************** * ****

IRF3a        CGCCAACTGAGGGAGCGATGGCTGTCAATCACATTTCTCGGGAATATCAACAGACTGCCA
IRF3b        CGCCAACTGAGGGAGCGATGGCTGTCAATCACATTTCTCGGGAATATCAACAGACTGCCA
IRF3c        CACCGACTGAGGGAGCGACAACTGCCAATCATAATTCTCCAGAATATCAACAGACTGCCA
             * ** *************   *** ****** * *****  *******************

IRF3a        CTCAACCACATTTAGGAGCTGTACCTGATCACACTGCCATTCCTGAGCCAAATCTGGCGA
IRF3b        CTCAACCACATTTAGGAGCTGTACCTGATCACACTGCCATTCCTGAGCCAAATCTGGCGA
IRF3c        CTCAACCACATTTAGGAGCTGTAACTGATCACACTGCCATTCCTGAGCCAAATCTGGCGA
             *********************** ************************************

IRF3a        CATTTTTTCGGATCAAGGTTTATTATAAAGGGAAAATGGTGATGGAGCAGTTGGTGGAGA
IRF3b        CATTTTTTCGGATCAAGGTTTATTATAAAGGGAAAATGGTGATGGAGCAGTTGGTGGAGA
IRF3c        CATTTTTTCAGATCAAGGTTTATTACAGAGGGAAAATGGTGATGGAGCAGTTGGTGGAGA
             ********* *************** * *******************************

IRF3a        ACGACTCTGGTTTCAGACTGATGTACCACAGAAACATGGATGAGTCAGGCCTGCAGGATG
IRF3b        ACGACTCTGGTTTCAGACTGATGTACCACAGAAACATGGATGAGTCAGGCCTGCAGGATG
IRF3c        ACGACTCTGGTTTCAGACTGGTGTACCAC------------GAGTCAGGCCTGCAGGACA
             ******************** ********            *****************

IRF3a        GTGCGGGTCTCCCAGTGGTCACTCTTCCTCCAGCTGAAGGAATGCCGGATCAAATGCAGA
IRF3b        GTGCGGGTCTCCCAGTGGTCACTCTTCCTCCAGCTGAAGGAATGCCGGATCAAATGCAGA
IRF3c        GTGCAGGTCTCCCAGTAGTCACTCTTCCTCCAGCTGAAGGAATACCGGATCAAGTGCAGA
             **** *********** ************************** ********* ******

IRF3a        CCAGGCTTACTAATGATATTCTGGATAATCTGGGTGGTCTGGAGATTCGGAGATCAGACG
IRF3b        CCAGGCTTACTAATGATATTCTGGATAATCTGGGTGGTCTGGAGATTCGGAGATCAGACG
IRF3c        CTGGGCATACTAATAATATTCTGGATAACCTGGGTGGTCTGGAGATTCGGAGATCAGACG
             *  *** ******* ************* *******************************

IRF3a        GAGTGATTCACGGTCACCGTTGGGGGTCCAGCAGGATCTACTGGGGTCTGTGCAAGCATG
IRF3b        GAGTGATTCACGGTCACCGTTGGGGGTCCAGCAGGATCTACTGGGGTCTGTGCAAGCATG
IRF3c        GAGTGATTTACGGTCACCGTTGGGGGTCCAGCAAGATCTACTGGGGTCAGTGTAAACATG
             ******** ************************ ************** *** ** ****

IRF3a        AAAGAAGCCAAACACCCAGAGAACTGTCCAAAAACACACCTCAACCCATTTACCTCATGA
IRF3b        AAAGAAGCCAAACACCCAGAGAACTGTCCAAAAACACACCTCAACCCATTTACCTCATGA
IRF3c        AAAGAAGCCAAACACCCAGAGAACTGTCCAAAAACACACCTCAACCCATTTACCTCATGA
             ************************************************************

IRF3a        AAGATTACATTTC-----------------------------------------------
IRF3b        AAGATTACATTTCAGGTGGTAGGCCGGTCAATTTTTTGGCCGAGGTCGATCTCAATAAAC
IRF3c        AAGATTACATTTC-----------------------------------------------
             *************

IRF3a        ----------------------------AGGTTTGATGCAGTTTATTCAGACAGGAGGTG
IRF3b        ATGGATGTCTGATGGTGTATCGTTCTGTAGGTTTGATGCAGTTTATTCAGACAGGAGGTG
IRF3c        ----------------------------AGGTTTGATGCAGTTTATGCAGACAGGAGGTG
                                         ****************** *************

IRF3a        AATCTCCTTCTTGCACTCTTTACTTCTTCCTGGGGGAGAAGTGGCCAGACCCTAAAATGA
IRF3b        AATCTCCTTCTTGCACTCTTTACTTCTTCCTGGGGGAGAAGTGGCCAGACCCTAAAATGA
IRF3c        AATCTCCTTCTTGCACTCTTTACTTCTTCCTGGGGGAGAAGTGGCCAGACCCTAAAATGA
             ************************************************************

IRF3a        AGCCTTGGGAGAAGAAACTCATCATGATTGAGGTTAATCTGACCGCACTGGAGTTTTTGA
IRF3b        AGCCTTGGGAGAAGAAACTCATCATGATTGAGGTTAATCTGACCGCACTGGAGTTTTTGA
IRF3c        AGCCTTGGGAGAAGAAACTCATCATGATTGAGGTTAATCTGACCGCATTGGAGTTTTTGA
             *********************************************** ************

IRF3a        AGTCTATGGCTGTTGAACAAGGAGCGTCTTCACTGCAGTCAGACGAGCTGCAGCTTTCCC
IRF3b        AGTCTATGGCTGTTGAACAAGGAGCGTCTTCACTGCAGTCAGACGAGCTGCAGCTTTCCC
IRF3c        AGTCTATGGCTGTTGAACAAGGAGCGTCTTCACTGCAGTCAGACGAGCTGCAGCTTTCCC
             ************************************************************

IRF3a        TGGAGCAAATGATGGAGCTCTGCTAAAAACAACCTCCTGGGTTAACCTGTTTACACTGTG
IRF3b        TGGAGCAAATGATGGAGCTCTGCTAA----------------------------------
IRF3c        TGGAGCAAATGATGGAGCTCTGCTG-----------------------------------
             ************************

IRF3a        GTCGTCCGGATCCTCTTCTGATTCTTTCTCCTCCATGGACAGATCTCTGAGCTCGGACAG
```

```
IRF3b         ------------------------------------------------------------
IRF3c         ------------------------------------------------------------

IRF3a         CGGCACCAGCAGACCCTCTTTATTCAGTCCCATCGGCTGAATCACATTATCCCTGTTTAA
IRF3b         ------------------------------------------------------------
IRF3c         ------------------------------------------------------------

IRF3a         GAAAAAACACAAGAAGGTTTGAGAACCTAACAAAGGATCCTTCTGGAAAAACTGAAACAA
IRF3b         ------------------------------------------------------------
IRF3c         ------------------------------------------------------------

IRF3a         TTGATTTGA
IRF3b         ---------
IRF3c         ---------
```

Figure S2: Multiple alignment of the protein sequences of zebrafish IRF3a, IRF3b and IRF3c-1. Identical residues are identified by stars, whereas similar residues are identified by single dots.

```
IRF3a          MTQAKPLFVPWLYEQIQSGRYPGVCWKNEDCTQFSIPWKHALRQDSNSDDVLIFKAWAQT
IRF3b          MTQAKPLFVPWLYEQIQSGRYPGVCWKNEDCTQFSIPWKHALRQDSNSDDVLIFKAWAQT
IRF3c-1        MTQAKPLFVPWLYEQIQSGRYPGVCWKNEDCTQFSIPWKHALRQDSNSDDVLIFKAWAQT
               ************************************************************

IRF3a          SAAGDGRLNGDPSVWKRNFRSALRAKGFKMISDKKNDGADPHKVYQFPSDPHSAASGSEG
IRF3b          SAAGDGRLNGDPSVWKRNFRSALRAKGFKMISDKKNDGADPHKVYQFPSDPHSA------
IRF3c-1        SAAGDGRLNGDPSVWKRNFRSALRAKGFKMISDKKNDGADPHKVYQFPSDPHSAASGSEG
               ******************************************************

IRF3a          SQETDISPHLYVDNVFTASFDLDQHLTGLHLQDPSARVHIILNPNDLYVDPGTQIYDFQA
IRF3b          -------------------------------------VHIILNPNDLYVDPGTQIYDFQA
IRF3c-1        SQETD-------------------------------------------------------


IRF3a          VVPIEAISSSDEGAVGGLGQISPTEGAMAVNHISREYQQTATQPHLGAVPDHTAIPEPNL
IRF3b          VVPIEAISSSDEGAVGGLGQISPTEGAMAVNHISREYQQTATQPHLGAVPDHTAIPEPNL
IRF3c-1        ------------------------------------------------------------

IRF3a          ATFFRIKVYYKGKMVMEQLVENDSGFRLMYHRNMDESGLQDGAGLPVVTLPPAEGMPDQM
IRF3b          ATFFRIKVYYKGKMVMEQLVENDSGFRLMYHRNMDESGLQDGAGLPVVTLPPAEGMPDQM
IRF3c-1        ------------------------------------------------------------

IRF3a          QTRLTNDILDNLGGLEIRRSDGVIHGHRWGSSRIYWGLCKHERSQTPRELSKNTPQPIYL
IRF3b          QTRLTNDILDNLGGLEIRRSDGVIHGHRWGSSRIYWGLCKHERSQTPRELSKNTPQPIYL
IRF3c-1        ------------------------------------------------------------

IRF3a          MKDYIS-------------------------GLMQFIQTGGESPSCTLYFFLGEKWPDPK
IRF3b          MKDYISGGRPVNFLAEVDLNKHGCLMVYRSVGLMQFIQTGGESPSCTLYFFLGEKWPDPK
IRF3c-1        ------------------------------------------------------------

IRF3a          MKPWEKKLIMIEVNLTALEFLKSMAVEQGASSLQSDELQLSLEQMMELC
IRF3b          MKPWEKKLIMIEVNLTALEFLKSMAVEQGASSLQSDELQLSLEQMMELC
IRF3c-1        -------------------------------------------------
```

Figure S3: Multiple alignment of the protein sequences of zebrafish IRF3a, IRF3b and IRF3c-2. Identical residues are identified by stars, whereas similar residues are identified by single dots.

```
IRF3a           MTQAKPLFVPWLYEQIQSGRYPGVCWKNEDCTQFSIPWKHALRQDSNSDDVLIFKAWAQT
IRF3b           MTQAKPLFVPWLYEQIQSGRYPGVCWKNEDCTQFSIPWKHALRQDSNSDDVLIFKAWAQT
IRF3c-2         ------MFI--------------IC-----------------------------------
                      :*:              :*

IRF3a           SAAGDGRLNGDPSVWKRNFRSALRAKGFKMISDKKNDGADPHKVYQFPSDPHSAASGSEG
IRF3b           SAAGDGRLNGDPSVWKRNFRSALRAKGFKMISDKKNDGADPHKVYQFP------------
IRF3c-2         ACAGAHRL------FQRHTQSL--------------------------------------
                :.**  **      ::*: .*

IRF3a           SQETDISPHLYVDNVFTASFDLDQHLTGLHLQDPSARVHIILNPNDLYVDPGTQIYDFQA
IRF3b           -------------------------------SDPHSAVHIILNPNDLYVDPGTQIYDFQA
IRF3c-2         ----KISPHLYVDNVFTASLDLDQHFTGLNLQDPSAHVYRILNPDDLYVDPGTQILDFQA
                                                .**: *: ****:********** ****

IRF3a           VVPIEAISSSDEGAVGGLGQISPTEGAMAVNHISREYQQTATQPHLGAVPDHTAIPEPNL
IRF3b           VVPIEAISSSDEGAVGGLGQISPTEGAMAVNHISREYQQTATQPHLGAVPDHTAIPEPNL
IRF3c-2         VVPIKAISSSDEGAVGGLGKISPTEGATTANHNSPEYQQTATQPHLGAVTDHTAIPEPNL
                ****:**************:******* :.** * **************.**********

IRF3a           ATFFRIKVYYKGKMVMEQLVENDSGFRLMYHRNMDESGLQDGAGLPVVTLPPAEGMPDQM
IRF3b           ATFFRIKVYYKGKMVMEQLVENDSGFRLMYHRNMDESGLQDGAGLPVVTLPPAEGMPDQM
IRF3c-2         ATFFQIKVYYRGKMVMEQLVENDSGFRLVYH----ESGLQDSAGLPVVTLPPAEGIPDQV
                ****.*****.*****************:**    ******.*************:***:

IRF3a           QTRLTNDILDNLGGLEIRRSDGVIHGHRWGSSRIYWGLCKHERSQTPRELSKNTPQPIYL
IRF3b           QTRLTNDILDNLGGLEIRRSDGVIHGHRWGSSRIYWGLCKHERSQTPRELSKNTPQPIYL
IRF3c-2         QTGHTNNILDNLGGLEIRRSDGVIYGHRWGSSKIYWGQCKHERSQTPRELSKNTPQPIYL
                **  **:*****************:*******.**** **********************

IRF3a           MKDYIS-------------------------GLMQFIQTGGESPSCTLYFFLGEKWPDPK
IRF3b           MKDYISGGRPVNFLAEVDLNKHGCLMVYRSVGLMQFIQTGGESPSCTLYFFLGEKWPDPK
IRF3c-2         MKDYIS-------------------------GLMQFMQTGGESPSCTLYFFLGEKWPDPK
                ******                         *****:***********************

IRF3a           MKPWEKKLIMIEVNLTALEFLKSMAVEQGASSLQSDELQLSLEQMMELC
IRF3b           MKPWEKKLIMIEVNLTALEFLKSMAVEQGASSLQSDELQLSLEQMMELC
IRF3c-2         MKPWEKKLIMIEVNLTALEFLKSMAVEQGASSLQSDELQLSLEQMMELC
                *************************************************
```
